# A Hospital-based Study on the Local Epidemiology of Pneumonia Including the Contribution of Legionella Pneumonia

**DOI:** 10.21315/mjms2020.27.6.8

**Published:** 2020-12-29

**Authors:** Albert Iruthiaraj Lourdesamy Anthony, Zarifah Zam, Narwani Hussin

**Affiliations:** 1 Respiratory Unit, Hospital Taiping, Taiping, Malaysia; 2 Microbiology Unit, Hospital Taiping, Taiping, Malaysia; 3 Clinical Research Centre, Hospital Taiping, Taiping, Malaysia

**Keywords:** epidemiology, pneumonia, microbial etiology, culture-negative, Legionella pneumonia

## Abstract

**Background:**

In real-life practice, only 20% of hospitalised pneumonia cases have an identified etiology. The usage of *Legionella* urine antigen test (LUAT) in developed nations revolutionised case detection rates. Accordingly, our objectives were to study the microbiological etiology for hospitalised pneumonia patients and the diagnosis of *Legionella* pneumonia.

**Methods:**

A prospective, observational single-centre study was conducted where all 504 cases that were consecutively admitted for pneumonia were enrolled. Blood and sputum samples obtained were used to identify pathogens using standard microbiological culture methods. The urine samples collected were tested using the Immunocatch^TM^ *Legionella* immunochromatographic (ICT) urine antigen test.

**Results:**

A microbiological diagnosis was only achieved in 104 cases (20.6%) and a Gram-negative infection predominance was observed. Culture-positive cases required longer hospitalisation (8.46 days versus 5.53 days; *P* < 0.001) and the higher usage of antipseudomonal antibiotics (23.1% versus 8.3%; *P* < 0.001). Only 3 cases (0.6%) were diagnosed with *Legionella* pneumonia.

**Conclusion:**

The local pathogen distribution is diverse compared to other regions. Culture-negative pneumonia is common and significantly differs from culture-positive pneumonia. *Legionella pneumophila* serotype 1 is not a common cause of pneumonia and LUAT did not help demystify the cause of culture-negative pneumonia.

## Introduction

Pneumonia is one of the most common causes of infectious disease death in the world and mortality is highest among patients who are hospitalised (1). It is also reputed to be one of the leading causes of hospitalisation, which has risen by 20%–50% for the past decade (2, 3). Incidence rates have varied in diverse populations from 1.3 to 14 cases per 1,000 population/year; the substantial burden placed on the healthcare system is heterogeneous due to demographic and geographical differences (4–7).

*Streptococcus pneumoniae* is frequently the most common cause of community-acquired pneumonia in Europe and other developed countries (8, 9). However, a few epidemiological studies in Asia have revealed the etiological diagnosis for patients hospitalised with pneumonia was only possible in 60% of cases in a research setting and only a dismal 20% in real-life practice due to the limited availability of specialised tests (10–12). For instance, in the USA and Europe, case detection rates for *Legionella* pneumonia were revolutionised with the usage of urine antigen testing at 97% and 79%, respectively (13, 14). Most cases hospitalised with pneumonia in Asia are usually dismissed as non-*Legionella* without appropriate testing due to the perception that this infection is relatively uncommon in this part of the world.

The primary objective of this study was to examine the local microbiological distribution according to etiology and to compare the demographic characteristics between cases with and without a microbiological etiology. The secondary objective of this study was to determine the incidence of *Legionella* pneumonia using the *Legionella* urine antigen test (LUAT) among patients hospitalised with pneumonia in a community hospital. In our centre, routine testing with LUAT is not conducted in real-life practice.

## Methods

### Study Population and Design

This study was conducted at Hospital Taiping, Taiping, Perak which has a bed capacity of 608 and a reputation as a community and regional teaching hospital in the state of Perak in Malaysia, serving a combined rural and urban population of 477,000. In this prospective, observational, single-centre study, which has a cross-sectional study design, 504 patients aged 18 years and older hospitalised for pneumonia were enrolled from September 2017 until May 2019. All cases consecutively admitted for pneumonia, irrespective of it being community or hospital acquired, were included. Eligible participants had acute symptoms of less than 2 weeks and radiological features compatible with pneumonia. All cases were defined as community acquired unless acquired after 48 h of hospitalisation or if the patient had significant healthcare contact within the last 3 months. Such cases were defined as hospital acquired. Exclusion criteria comprised patients suspected to have active tuberculosis and patients unwilling or unable to give consent.

### Sample Size Calculation

Sample sizes were calculated using the single proportion formula. We needed at least 457 cases to be included to achieve a 2% (0.02) precision in estimating the expected incidence of 5% (0.05). After considering a 10% dropout, we decided to include 504 patients. The level of confidence was set at 95%.

### Microbiological Analysis and Data Collection

Demographic, clinical and laboratory information was retrieved at the time of admission from medical records. Demographic information included age, sex and possible risk factors for pneumonia. Clinical information included reported symptoms retrieved from respective patients and documentation in medical records by their respective clinicians. Laboratory blood tests and blood cultures were obtained for all patients before starting antibiotics. Meanwhile, most sputum samples for cultures were obtained before antibiotics commenced (> 85% of cases) and as for the rest, sputum was induced via nebulised hypertonic saline and obtained within 24 h of admission and antibiotic prescription. All information gathered was listed in the standardised case report form, which was used for data analysis. A viral etiology was not pursued in this study, as it was not routinely tested in our centre. The case report forms were updated for possible complications, changes in antibiotics and mortalities within 30 days of hospitalisation.

Eligible participants underwent testing for *Legionella pneumophila* serogroup 1 urine antigen using the Immunocatch^TM^ *Legionella* immunochromatographic (ICT) urine antigen test (Eiken Chemical Co. Ltd., Japan) after being identified in the emergency department and enrolled into the study. The urine specimens were evaluated in our microbiology lab. The state health department was notified of cases assessed positive via fax using the standard notification form within 24 h of diagnosis. The patient and the responsible clinician managing the respective cases were notified of the results, but the clinical interpretation of these results was left at the discretion of treating clinicians.

A second urine specimen for positive cases was sent to a tertiary centre via the microbiology laboratory for verification purposes. For verifying diagnoses, Binax^TM^ *Legionella* enzyme immuno assay (EIA) urine antigen test (Alere Inc., USA) was used instead of the gold standard test of cultures on specialised media/buffered-charcoal yeast extract (BCYE) plates, as the latter is not available nationwide. The case is considered confirmed if the *Legionella* urine EIA test is positive.

### Statistical Analysis

The statistical analysis was performed using IBM Statistical Package for the Social Sciences (SPSS) for Windows, version 21.0. Armonk, NY, USA. Descriptive statistics including patient demographics, clinical and laboratory parameters were presented using mean (standard deviation) for normally distributed variables and frequency (percentage) for categorical variables. Frequency comparisons were conducted via Pearson’s Chi-square test or Fisher’s exact test for categorical variables and an independent *t*-test for continuous variables. Univariable analyses showed distinctions between characteristics of interest. The results were considered significant if the *P*-value was less than 0.05.

## Results

### Patient Characteristics

A total of 504 patients were enrolled in the study. Table 1 shows the characteristics of patients hospitalised with pneumonia and Table 2 displays the distinctions between characteristics for older and younger patients with pneumonia. There were 407 (80.8%) patients classified as having community-acquired pneumonia (CAP) and 97 (19.2%) with hospital-acquired pneumonia (HAP). Overall, 45% of them were more than 65 years of age.

### Pneumonia Etiology

A microbiological diagnosis was only achieved in 104 cases (20.6%). Sputum culture and blood culture yields were 16.3% and 3.7%, respectively. Table 3 reveals the pathogen distribution for patients with CAP and HAP. Among the 407 patients diagnosed with CAP, Gram-negative infection predominance was observed. Paradoxically, *Pseudomonas aeruginosa* was detected in 12 cases of CAP. However, all these cases had pre-existing chronic lung disease or chronic kidney disease. Patients with HAP were also more likely to be infected with Gram-negative bacilli infection. *Legionella pneumophila* serotype 1 was detected only in 3 cases of CAP using ICTs, giving it a yield of 0.6%. All three specimens were also validated as positive by urine EIA tests and no other bacteria were isolated in their blood and sputum cultures. The etiology of pneumonia in this study remains unknown in 400 cases (79.4%).

Table 4 reveals the disparities in the characteristics of hospitalised pneumonia patients with and without an identified microbiological etiology. The mean length of hospitalisation was 2.93 days (8.46 days versus 5.53 days; *P* < 0.001) longer for patients with positive cultures.

## Discussion

To the best of our knowledge, this research was one of the first hospital-based studies to comprehensively evaluate the epidemiological factors of patients hospitalised with pneumonia in Malaysia, satisfying all the following: i) a prospective design; ii) real-world data involving more than 500 consecutive patients; and iii) a comprehensive evaluation of *Legionella pneumophila* as a possible etiology of pneumonia using ICT urine antigen testing.

Overall, our cohort of patients hospitalised with pneumonia was mostly middle-aged women who presented with a typical triad of symptoms of fever, cough and shortness of breath. However, patients with HAP were mostly older men who presented with shortness of breath as the predominant symptom frequently with pre-existing chronic lung disease or chronic kidney disease. Patients with HAP from the present study were far more ill, requiring vasopressors, longer hospitalisation and longer duration of antibiotics, as well as higher usage of antipseudomonal antibiotics and carbapenem. The 30-day mortality rate for HAP patients (7.2%) was also more than twice that of the 30-day mortality rate at 3%. Although it is well recognised that patients hospitalised with HAP suffer from disproportionately higher mortality and morbidity, the data on the incidences and demographic features of CAP and HAP patients are limited (15, 16). The overall 30-day mortality rate and the mean length of hospitalisation from the present study were remarkably lower compared to previous studies, which suggests that our patients overall had milder disease severity and presumably a better response to the antimicrobials instituted (17, 18).

Because of global improvements in life expectancy expanding the population of the elderly, pneumonia is reputed as one of the most common reasons for hospitalisation for persons aged 65 years and above (19). A significant minority (45%) of our study population belonged to this age category and they had more co-morbidities than younger patients. Smoking and pre-existing lung disease have been identified as predictors of pneumonia in this group of patients, as shown in a previous study (20). However, the mechanisms of how co-morbidities predispose elderly patients to pneumonia remain unclear (21). Although intensive vasopressor and respiratory support requirements were lower, the 30-day mortality rate was eightfold higher in the elderly population. The severity of pneumonia in this population can often be underestimated in the absence of shock or respiratory distress. Atypical and occult presentations can lead to diagnostic and treatment delays. Furthermore, a poor outcome is likely when elderly patients develop severe diseases (22).

For CAP and HAP, Gram-negative organisms were discovered to be the most common etiology for hospitalised culture-positive pneumonia patients. Although the spectrum of etiological pathogens for pneumonia has been reported to be diverse between studies, Liam et al. (11) suggested that the pathogen distribution in hospitalised patients with pneumonia in Malaysia is substantially variable compared to Western countries. Pneumococcal disease could have been underestimated in the present study, as we relied solely on sputum and blood cultures besides LUAT to extrapolate the etiology of pneumonia. The utility of the pneumococcal urine antigen test, as in other studies, may have improved the detection of *Streptococcus pneumoniae*, especially in patients who could not produce sputum or in those who were not bacteremic (23). Furthermore, a viral etiology was not pursued, which could explain the high number of cases (79.4%) without etiologies in the present study. We agree that the variations in pathogen distribution largely depend on geographic characteristics, variances in study populations and investigations used to identify the causal pathogen.

We observed that culture-negative pneumonia is common, as demonstrated in our study, but is poorly understood. Such patients in our study were younger and required shorter hospitalisations as well as durations of antibiotics with a lower usage of antipseudomonal antibiotics. Similarly, an observational study that exclusively included intensive care unit (ICU) patients reported that patients with culture-negative sepsis had milder disease severity and shorter length of hospitalisations (24). Although there are overt disparities in characteristics between culture-negative and culture-positive pneumonia, research on the outcomes of these patients is relatively scarce. Cultures may lack the sensitivity to identify all bacteria and patients with culture-negative pneumonia may have a lower bacterial burden, further limiting the capacity of cultures to yield a positive result (25, 26). It may be hypothesised that viruses could cause pneumonia in a significant number of our culture-negative patients but testing for such is not pursued in our setting and is beyond the scope of our research (27).

Globally, 1%–5% of pneumonia is caused by *Legionella* species, which challenges public health authorities (28). Since it is impossible to distinguish the etiology of pneumonia clinically and the use of LUAT has revolutionised case detection rates in Europe and USA (13, 14), we explored the epidemiology of *Legionella* pneumonia in our local practice. Risk factors for *Legionella* pneumonia include cigarette smoking, chronic lung disease, older age, diabetes and immunosuppression (29). A large proportion of our patients were 65 years old and older with such co-morbidities. The low number of cases detected support the findings from studies in Singapore, Japan and Iran, which showed a far lower incidence of *Legionella* pneumonia compared to Western countries (10, 30, 31). Hence, *Legionella* is an uncommon etiological cause of pneumonia compared to other microorganisms and it is not cost-effective to screen all pneumonia patients requiring hospitalisation in a low- to medium-resource setting like ours.

*Legionella* urine antigen ICT testing has reported optimum sensitivity that ranges between 76% and 86% for cases of *Legionella pneumophila* serogroup 1 and a specificity that approaches 100% but is rarely done in our practice due to the lack of availability of this test regionally (32, 33). The ICT assay is an ideal tool for point-of-care testing and screening for *Legionella* infection, as it does not require special laboratory equipment and results can be obtained within minutes. The sensitivity of the ICT assays for both the Immunocatch^TM^ LUAT ICT test and the commercially available competitor, BinaxNOW^®^ LUAT ICT test (Alere Inc., USA) for the *Legionella pneumophila* serogroup 1 was 80%, and the specificity was 97% with a 98% overall agreement between the two tests. Comparison between the ICT assays and the urine EIA test showed 98% overall agreement and a specificity of 100% (33–35). This discovery has allowed a five-day reduction for the delay between the onset of illness and notification, as well as facilitated decreasing mortality trends in developed nations. Diagnoses with cultures have their limitations. A successful culture requires selective media, expertise and prolonged incubation periods. Serological tests may require the demonstration of paired seroconversions obtained two weeks apart and have been less popular in recent years. In routine clinical practice, legionellosis is rarely proven by culture, whereas the detection of urinary antigens is now common (13, 14, 32).

From our study, only 3 out of 504 cases (0.6%) were detected to be positive for *Legionella* from LUAT ICT testing and subsequently verified with the *Legionella* urine EIA test. These three cases were treated with intravenous azithromycin for 10 days at the discretion of their treating clinicians as per local and international antibiotic guidelines. Repeat testing with LUAT ICT testing or EIA testing was not pursued for these cases due to the limited availability of test kits. However, we do agree that repeat testing during or after treatment may have given guidance to the treatment response and efficacy of antimicrobials. All three cases were discharged home within 10 days of hospitalisation after showing clinical recovery. However, one of the patients, a 40-year-old man with pre-existing cerebrovascular disease, was re-admitted and died within 30 days of the initial hospitalisation. Mortality audit at the hospital level concluded that death was due to a recurring cerebrovascular accident. For the positive cases, public health authorities could not identify the source of the infection from their respective residences.

### Study Limitations

The present study is not without important limitations. First, ours was a single-centre study with an observational design. Therefore, the findings may not be applicable to other centres, as subjects were recruited from a limited region in one country. Despite the prospective analysis, the nature of the observational study design could not eliminate potential bias. Therefore, with the presence of unadjusted confounders, we could only suggest associations between variables of interest. Second, given that we did not pursue additional testing for a non-bacterial etiology to establish the diagnosis of pneumonia, our findings cannot be extrapolated to patients with non-bacterial causes of pneumonia. Last, the management decisions of the patients were left to the discretion of clinicians without interference from investigators. Undoubtedly, there could have been discordance between clinicians despite their best efforts to adhere to guidelines and this might have affected the overall outcomes of our patients.

## Conclusion

In conclusion, we discovered that patients with HAP and culture-positive pneumonia had higher severity of illness requiring longer hospitalisation. Mortality was also significantly remarkable in patients with HAP and persons aged 65 years and older. In contrast to previous studies, Gram-negative organisms were found to be the most common etiology of pneumonia. In addition, the current study suggests that *Legionella pneumophila* serogroup 1 is not a common cause of pneumonia in our locality and is also not the etiology of the large numbers of culture-negative pneumonia cases identified. A larger multicentre research is needed to explain the distinctions observed in the epidemiology and outcomes in patients with culture-positive versus culture-negative pneumonia.

## Figures and Tables

**Table 1:** Characteristics of patients hospitalised with pneumonia

Characteristics	Total (*n* = 504)	Community (*n* = 407)	Nosocomial (*n* = 97)	*P*-value
	Frequency (%)	Frequency (%)	Frequency (%)	
Age (year)*	59.79 (16.46)	59.10 (16.65)	62.66 (15.42)	0.056^a^
Male	228 (45.2)	177 (43.5)	51 (52.6)	0.106^b^
Symptoms				
Fever	350 (69.4)	293 (72.0)	57 (58.8)	0.011^b^
Cough	440 (87.3)	360 (88.5)	80 (82.5)	0.112^b^
Shortness of breath	380 (75.4)	299 (73.5)	81 (83.5)	0.039^b^
Chest pain	77 (15.3)	59 (14.5)	18 (18.6)	0.318^b^
Anorexia	120 (23.8)	104 (25.6)	16 (16.5)	0.060^b^
Diarrhea	29 (5.8)	26 (6.4)	3 (3.1)	0.210^b^
Co-morbidities				
Chronic lung disease	117 (23.2)	76 (18.7)	41 (42.3)	< 0.001^b^
Kidney disease	28 (5.6)	17 (4.2)	11 (11.3)	0.006^b^
Diabetes	174 (34.5)	143 (35.1)	31 (32.0)	0.554^b^
Malignancy/cancer	15 (3.0)	11 (2.7)	4 (4.1)	0.504^c^
Hypertension	212 (42.1)	179 (44.0)	33 (34.0)	0.074^b^
Smoking status				
Current	88 (17.5)	79 (19.4)	9 (10.2)	< 0.001^b^
Former	103 (20.4)	70 (17.2)	33 (34.0)	
ICU admission	33 (6.5)	25 (6.1)	8 (8.2)	0.451^b^
Mechanical ventilation	30 (6.0)	22 (5.4)	8 (8.2)	0.288^b^
Shock with vasopressors	36 (7.1)	23 (5.7)	13 (13.4)	0.008^b^
Length of stay (day)*	6.14 (4.14)	5.76 (3.69)	7.71 (5.37)	0.001^a^
30-day mortality	15 (3.0)	8 (2.0)	7 (7.2)	0.013^c^
Antibiotics				
Combined regimen^d^	230 (45.6)	209 (51.4)	21 (21.6)	< 0.001^b^
Antipseudomonal antibiotics	57 (11.3)	27 (6.6)	30 (30.9)	< 0.001^b^
Carbapenem	13 (2.6)	5 (1.2)	8 (8.2)	0.001^c^
Duration of antibiotics (day)*	6.59 (2.47)	6.47 (2.37)	7.06 (2.81)	0.035^a^

Notes: Values are presented as mean (%) for binary variables unless indicated;

* Values are presented as mean (standard deviation) for continous variables; *P*-values are calculated, where appropriate, using the independent *t*-test^a^, Chi-square test^b^ and Fisher’s exact test^c^; β-lactam and macrolide combination^d^

**Table 2:** Differences of characteristics between older and younger patients hospitalised with pneumonia

Characteristics	Age ≥ 65 years (*n* = 227)	Age < 65 years (*n* = 277)	*P*-value
	Frequency (%)	Frequency (%)	
Comorbidities			
Chronic lung disease	73 (32.2)	44 (15.9)	< 0.001^b^
Kidney disease	14 (6.2)	14 (5.1)	0.587^b^
Diabetes	88 (38.8)	86 (31.0)	0.070^b^
Malignancy/cancer	6 (2.6)	9 (3.2)	0.690^b^
Hypertension	118 (52.0)	94 (33.9)	< 0.001^b^
Current and former smokers	102 (44.9)	89 (32.1)	0.003^b^
Recent hospitalisation	49 (21.6)	41 (14.8)	0.048^b^
ICU admission	6 (2.6)	27 (9.7)	0.001^b^
Mechanical ventilation	8 (3.5)	22 (7.9)	0.037^b^
Shock with vasopressors	14 (6.2)	22 (7.9)	0.441^b^
Length of stay (day)*	6.04 (3.91)	6.22 (4.32)	0.618^a^
30-day mortality	13 (5.7)	2 (0.7)	0.001^b^
Carbapenem and antipseudomonal antibiotic use	36 (15.9)	33 (11.9)	0.200^b^
Duration of antibiotics (day)*	6.41 (2.50)	6.73 (2.43)	0.154^a^

Notes: Values are presented as mean (%) for binary variables unless indicated;

* Values are presented as mean (standard deviation) for continous variables; *P*-values are calculated, where appropriate, using the independent *t*-test^a^ and Chi-square test^b^

**Table 3:** Pathogen distribution for patients hospitalised with CAP and HAP

Organism	CAP (*n* = 70/407)			Organism	HAP (*n* = 34/97)		
	*n* (%)	Sputum (*n*)	Blood (*n*)		*n* (%)	Sputum (*n*)	Blood (*n*)
*Klebsiella pneumoniae*	25 (6.1)	21	4	*Acinetobacter baumanii*	9 (9.3)	9	–
*Pseudomonas aerugonisa*	12 (2.9)	12	–	*Pseudomonas aerugonisa*	6 (6.2)	5	1
*Moraxella* sp.	10 (2.5)	10	–	*Klebsiella pneumoniae*	5 (5.1)	4	1
*Haemophilus* sp.	5 (1.2)	4	1	*ESBL Enterobacteriaceae*^b^	4 (4.1)	2	2
*Enterobacter* sp.	4 (1.0)	2	2	*Enterobacter* sp.	3 (3.1)	3	–
*Burkholderia pseudomallei*	3 (0.7)	1	2	*Proteus* sp.	2 (2.1)	2	–
*Escherichia coli*	3 (0.7)	1	2	*MSSA*^a^	2 (2.1)	2	–
*Legionella pneumophila*	3 (0.7)	–	–	*Escherichia coli*	1 (1.0)	–	1
*Streptococcus pneumoniae*	3 (0.7)	1	2	*Stenotrophomonas maltophilia*	1 (1.0)	1	–
MSSA^a^	2 (0.5)	2	–	*Streptococcus pneumoniae*	1 (1.0)	1	–

Notes:

a MSSA = methicillin-sensitive *Staphylococcus aureus*;

b ESBL = extended-spectrum β-lactam *Klebsiella pneumoniae* (3 cases) and *Escherichia coli* (1 case)

**Table 4:** Differences in characteristics of hospitalised pneumonia patients with and without a microbiological etiology

Characteristics	Undetected etiology (*n* = 400)	Confirmed etiology (*n* = 104)	*P*-value
	Frequency (%)	Frequency (%)	
Age (year)*	58.94 (16.74)	63.06 (14.99)	0.024^a^
Gender			
Male	177 (44.3)	51 (49.0)	0.382^b^
Comorbidities			
Chronic lung disease	83 (20.8)	34 (32.7)	0.011^b^
Kidney disease	23 (5.8)	5 (4.8)	0.709^b^
Diabetes	133 (33.3)	41 (39.4)	0.239^b^
Hypertension	164 (41.0)	48 (46.2)	0.343^b^
Intensive care unit (ICU) admission	25 (6.3)	8 (7.7)	0.597^b^
Mechanical ventilation	22 (5.5)	8 (7.7)	0.402^b^
Shock with vasopressors	24 (6.0)	12 (11.5)	0.055^b^
Length of stay (day)*	5.53 (3.54)	8.46 (5.30)	< 0.001^a^
30-day mortality	10 (2.5)	5 (4.8)	0.225^b^
Antibiotics			
Combined regimen^c^	192 (48.0)	38 (36.5)	0.037^b^
Antipseudomonal	33 (8.3)	24 (23.1)	< 0.001^b^
Carbapenem	8 (2.0)	5 (4.8)	0.119^b^
Duration of antibiotics (day)*	6.05 (1.52)	8.65 (3.92)	< 0.001^a^
Sepsis parameters			
Total white cell count (×10^9^/L)*	13.71 (6.06)	15.28 (6.68)	0.023^a^
C-reactive protein (mg/L)*	19.00 (64.30)	69.05 (171.60)	< 0.001^a^

Notes: Values are presented as mean (%) for binary variables unless indicated;

* Values are presented as mean (standard deviation) for continous variables; *P*-values are calculated, where appropriate, using independent *t*-test^a^ and Chi-square test^b^; β-lactam and macrolide combination^c^

## References

[1] Lozano R, Naghavi M, Foreman K, Lim S, Shibuya K, Aboyans V (2012). Global and regional mortality from 235 causes of death for 20 age groups in 1990 and 2010: a systematic analysis for the Global Burden of Disease Study 2010. Lancet.

[2] Liu K, Lee GC (2019). Healthcare utilisation and cost expenditures for pneumonia in individuals with diabetes mellitus in the USA. Epidemol Infect.

[3] Fry AM, Shay DK, Holman RC, Curns AT, Anderson LJ (2005). Trends in hospitalizations for pneumonia among persons aged 65 years or older in the United States, 1988–2002. JAMA.

[4] Woodhead MA, Macfarlane JT, McCracken JS, Rose DH, Finch RG (1987). Prospective study of etiology and outcome of pneumonia in the community. Lancet.

[5] Almirall J, Bolibar I, Vidal J, Sauca G, Coll P, Niklasson B (2000). Epidemiology of community-acquired pneumonia in adults: a population-based study. Eur Respir J.

[6] Khan F, Owens MB, Restrepo M, Povoa P, Martin-Loeches I (2017). Tools for outcome prediction in patients with community acquired pneumonia. Expert Rev Clin Pharmacol.

[7] Tong S, Amand C, Kieffer A, Kyaw MH (2018). Trends in healthcare utilization and costs associated with pneumonia in the United States during 2008–2014. BMC Health Serv Res.

[8] Geno KA, Gilbert GL, Song JY, Skovsted IC, Klugman KP, Jones C (2015). Pneumococcal capsules and their types: past, present, and future. Clin Microbiol Rev.

[9] Welte T, Torres A, Nathwani D (2012). Clinical and economic burden of community-acquired pneumonia among adults in Europe. Thorax.

[10] Naderi H, Sheybani F, Sarvghad M, Meshkat Z, Nooghabi MJ (2015). Etiological diagnosis of community-acquired pneumonia in adult patients: a prospective hospital-based study in Mashhad, Iran. Jundishapur J Microbiol.

[11] Liam CK, Lim KH, Wong CM (2001). Community-acquired pneumonia in patients requiring hospitalization. Respirology.

[12] Marrie TJ, Basow DS, Waltham MA (2012). Epidemiology, pathogenesis, and microbiology of community-acquired pneumonia in adults. Uptodate.

[13] Levcovich A, Lazarovitch T, Moran-Gilad J, Peretz C, Yakunin E, Valinsky L (2016). Complex clinical and microbiological effects on Legionnaires’ disease outcome: a retrospective cohort study. BMC Infect Dis.

[14] Formica N, Yates M, Beers M, Carnie J, Hogg G, Ryan N (2001). The impact of diagnosis by legionella urinary antigen test on the epidemiology and outcomes of Legionnaires’ disease. Epidemiol Infect.

[15] Ewing S, Welte T, Chastre J, Torres A (2010). Rethinking the concepts of community-acquired and healthcare-associated pneumonia. Lancet Infect Dis.

[16] Micek ST, Kollef KE, Reicheley RM, Roubinian N, Kollef MH (2007). Healthcare-associated pneumonia and community-acquired pneumonia: a single-center experience. Antimicrob Agents Chemother.

[17] Olga OG, Angel VC, Cinta DD, Victoria A, Maxenchs M, Montserrat G (2008). The burden of community-acquired pneumonia in the elderly: the Spanish EVAN-65 study. BMC Pub Health.

[18] Carratalà J, Mykietiuk A, Fernández-Sabé N, Suarez C, Dorca J, Verdaguer R (2007). Healthcare-associated pneumonia requiring hospital admission: epidemiology, antibiotic therapy, and clinical outcomes. Arch Intern Med.

[19] Centers for Disease Control and Prevention (2013). Trends in aging – United States and worldwide. MMWR Morb Mortal Wkly Rep.

[20] Jackson M, Nelson J, Jackson L (2009). Risk factors for community-acquired pneumonia in immunocompetent seniors. J Am Geriatr Soc.

[21] Donowitz G, Cox H (2007). Bacterial community-acquired pneumonia in older patients. Clin Geriatr Med.

[22] Metlay J, Schulz R, Li Y, Singer D, Marrie T, Coley C (1997). Influence of age on symptoms at presentation in patients with community-acquired pneumonia. Arch Intern Med.

[23] Sorde R, Falco V, Lowak M, Domingo E, Ferrer A, Burgos A (2011). Current and potential usefulness of pneumococcal urinary antigen detection in hospitalized patients with community-acquired pneumonia to guide antimicrobial therapy. Arch Intern Med.

[24] Phua J, Ngerng W, See K, Tay C, Kiong T, Lim H (2013). Characteristics and outcomes of culture-negative versus culture-positive severe sepsis. Crit Care.

[25] Lever A, Mackenzie I (2007). Sepsis: definition, epidemiology, and diagnosis. BMJ.

[26] Lisboa T, Waterer G, Rello J (2010). We should be measuring genomic bacterial load and virulence factors. Crit Care Med.

[27] Choi SH, Hong SB, Ko GB, Lee Y, Park HJ, Park SY (2012). Viral infection in patients with severe pneumonia requiring intensive care unit admission. Am J Respir Crit Care Med.

[28] Diederen BMW, Kluytmans JAJW, Vandenbroucke-Grauls CM, Peeters MF (2008). Utility of real-time PCR for diagnosis of Legionnaires’ disease in routine clinical practice. J Clin Microbiol.

[29] Farnham A, Alleyne L, Cimini D, Balter S (2014). Legionnaires’ disease incidence and risk factors, New York, USA, 2002–2011. Emerg Infect Dis.

[30] Lam MC, Ang LW, Tan AL, James L, Goh KT (2011). Epidemiology and control of Legionellosis, Singapore. Emerg Infect Dis.

[31] Ishida T (2000). Etiology of community-acquired pneumonia among adult patients in Japan. Jpn J Antibiot.

[32] Touray S, Newstein MC, Lui JK, Harris M, Knox K (2014). *Legionella pnuemophilia* cases in a community hospital: a 12 month retrospective review. SAGE Open Med.

[33] Fields BS, Benson RF, Besser RE (2002). *Legionella* and Legionnaires’ disease: 25 years of investigation. Clin Microbiol Rev.

[34] Yamaguchi I, Kinoshita I, Saito T (2015). Usefulness evaluation of immunochromatography reagent for urinary Legionella antigen detection; comparing the performance of a new reagent and 3 existing reagents. Japanese J Med Technology.

[35] Congestrì F, Morotti M, Vicari R, Pedna MF, Sparacino M, Torri A (2019). Comparison of the novel immunocatch *Legionella* test with Sofia *Legionella* FIA assay and with BinaxNOWLegionella card assay for detection of *Legionella pneumophila* (serogroup 1) antigen in urine samples. J Clin Microbiol.

